# Mahanine restores *RASSF1A* expression by down-regulating DNMT1 and DNMT3B in prostate cancer cells

**DOI:** 10.1186/1476-4598-12-99

**Published:** 2013-08-30

**Authors:** Soumik Agarwal, Karishma S Amin, Shankar Jagadeesh, Gokul Baishay, Paruchuri G Rao, Nabin C Barua, Samir Bhattacharya, Partha P Banerjee

**Affiliations:** 1 Department of Biochemistry and Molecular & Cellular Biology, Georgetown University Medical Center, Washington, DC 20057, USA; 2 Natural Product Chemistry Division, North-East Institute of Science & Technology, Jorhat, Assam 785006, India; 3 Cellular and Molecular Endocrinology Laboratory, Centre for Advanced Studies in Zoology, School of Life Science, Visva-Bharati University, Santiniketan 731235, India; 4 Current address: ACell, Inc., Columbia, MD, USA

**Keywords:** Epigenetic silencing, RASSF1A, Tumor suppressor gene, DNMTs, Prostate cancer

## Abstract

**Background:**

Hypermethylation of the promoter of the tumor suppressor gene *RASSF1A* silences its expression and has been found to be associated with advanced grade prostatic tumors. The DNA methyltransferase (DNMT) family of enzymes are known to be involved in the epigenetic silencing of gene expression, including *RASSF1A,* and are often overexpressed in prostate cancer. The present study demonstrates how mahanine, a plant-derived carbazole alkaloid, restores *RASSF1A* expression by down-regulating specific members of the DNMT family of proteins in prostate cancer cells.

**Results:**

Using methylation-specific PCR we establish that mahanine restores the expression of *RASSF1A* by inducing the demethylation of its promoter in prostate cancer cells. Furthermore, we show that mahanine treatment induces the degradation of DNMT1 and DNMT3B, but not DNMT3A, via the ubiquitin-proteasome pathway; an effect which is rescued in the presence of a proteasome inhibitor, MG132. The inactivation of Akt by wortmannin, a PI3K inhibitor, results in a similar down-regulation in the levels DNMT1 and DNMT3B. Mahanine treatment results in a decline in phospho-Akt levels and a disruption in the interaction of Akt with DNMT1 and DNMT3B. Conversely, the exogenous expression of constitutively active Akt inhibits the ability of mahanine to down-regulate these DNMTs, suggesting that the degradation of DNMT1 and DNMT3B by mahanine occurs via Akt inactivation.

**Conclusions:**

Taken together, we show that mahanine treatment induces the proteasomal degradation of DNMT1 and DNMT3B via the inactivation of Akt, which facilitates the demethylation of the *RASSF1A* promoter and restores its expression in prostate cancer cells. Therefore, mahanine could be a potential therapeutic agent for advanced prostate cancer in men when *RASSF1A* expression is silenced.

## Background

Prostate cancer is the most common non-cutaneous malignancy in men in the Western world. The etiology of prostate cancer is not well defined; however, the inhibition of various tumor suppressor genes and concomitant activation of oncogenes is a frequent occurrence in most cancers, including prostate cancer. DNA hypermethylation of promoters at CpG sequences often sterically hinders the binding of transcription factors, thereby represses gene transcription [1]. The transfer of a methyl group to DNA at the fifth carbon position of cytosine residues by the DNA methyltransferase (DNMT) family of enzymes is one of the most common events for the establishment of epigenetic program [2]. Three active DNMTs have been identified so far in mammalian cells; DNMT1 (Gene ID: 1786), DNMT3A (Gene ID: 1788), and DNMT3B (Gene ID: 1789). DNMT1 is the most abundant and methylates hemimethylated CpG di-nucleotides in the mammalian genome during DNA replication in a number of different cancer types [3]. DNMT3A and DNMT3B are methyltransferases involved in de novo methylation of DNA following replication [4].

Numerous reports have demonstrated the overexpression of DNMT1 in lung, hepatocellular, acute and chronic myelogenous leukemia, colorectal, gastric, breast and prostate cancers [5-11]. Gravina and associates have shown that the hormone resistant prostate cancer phenotype is associated with an increase in DNMT expression and activity [12]. The majority of gene silencing induced by DNA hypermethylation in prostate cancer can be attributed to DNMT activity [13]. Since promoter hypermethylation results in the silencing of several tumor suppressor genes in cancer cells, the reactivation of these genes by demethylation of their promoters is a feasible approach to cancer therapy. DNMT inhibitors, such as 5-aza-deoxycytidine and 5-aza-2’-deoxycitidine, have been used extensively in clinical trials for cancer treatment. These nucleoside analogues bind covalently to the DNMTs and irreversibly inhibit their function, leading to the demethylation of silenced promoters and subsequently, the activation of gene expression. However, studies in rodent cell lines and bacteria have indicated that these azacitidine-DNMT adducts are toxic and mutagenic if not repaired [14-16]. In addition, these compounds are unstable in neutral aqueous solution and disintegrate to yield more stable analogues such as 5, 6-dihydro-5-azacytidine and 5-fluoro-2’-deoxycytidine [17], that have been shown to have toxicity related issues in clinical trials [18,19]. Therefore, there is an urgent need for the development of new drugs that will target DNMT with low toxicity.

Ras-association domain family 1A (*RASSF1A;* Gene ID: 11186) gene has been found to be the most frequently methylated gene described thus far in human cancers [20,21]. Hypermethylation of the promoter of *RASSF1A* gene at its CpG-island has been observed in 70% of prostate cancers [22,23]. Since the restoration of *RASSF1A* expression in tumor cell lines impairs tumorigenicity [22,24], factors that restore *RASSF1A* expression have immense importance in preventing tumor growth. We demonstrated earlier that mahanine induces *RASSF1A* gene expression in a diverse range of cancer cell types, including epidermoid, lung, pancreatic, colon, breast, ovarian and prostate cancer cells [25]. Although we showed a decline in DNMT activity upon mahanine treatment, the scope of our study did not include establishing a causative effect of DNMT inhibition on *RASSF1A* re-expression by mahanine, and this mechanism remains to be explored.

The cellular levels of DNMTs in mammalian cells can be regulated by transcriptional events or posttranslational modifications of enzymes which ultimately affect the catalytic activity and degradation of the DNMT proteins [26]. DNMT1 is known to be phosphorylated at several serine and threonine residues under physiological conditions. Sun and associates have reported that Akt enhances DNMT1 protein stability by inhibition of its ubiquitin–proteasome mediated degradation [27]. Recently it has been demonstrated that Akt1 directly interacts and phosphorylates Ser143 of DNMT1 to increase its stability [28].

In the present study, we sought to establish the mechanism by which mahanine inactivates DNMTs and thereby restores *RASSF1A* expression in prostate cancer cells. We show that mahanine induces the degradation of DNMT1 and DNMT3B via the ubiquitin-proteasome mediated pathway in both androgen-responsive LNCaP and androgen receptor-negative PC3 human prostate cancer cells. Interestingly, our data suggests that the inactivation of Akt by mahanine treatment is involved in inducing DNMT degradation, thereby restoring the expression of the epigenetically silenced tumor suppressor gene *RASSF1A*.

## Results

### Mahanine demethylates *RASSF1A* promoter and restores its expression in prostate cancer cells

**Figure 1:**
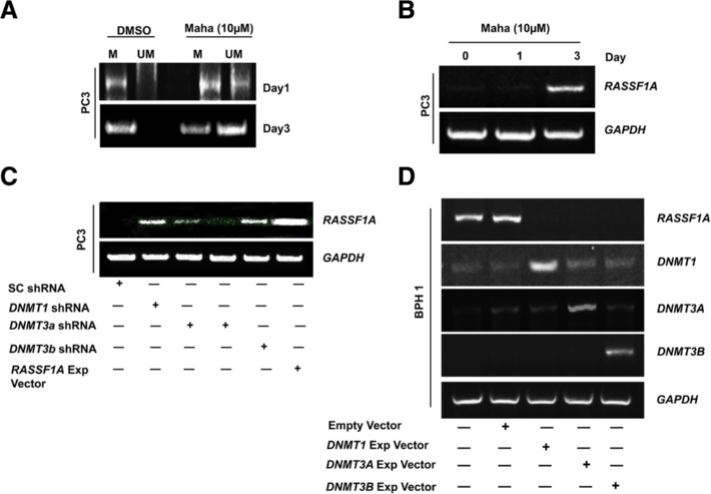
**Mahanine restores *RASSF1A *expression by demethylating its promoter and all three DNMTs control *RASSF1A *expression. A**. PC3 cells were treated with DMSO control or 10 μM mahanine for 1 and 3 days. Methylation-specific PCR was performed to detect the methylated (M) and un-methylated (UM) status of *RASSF1A* promoter. **B**. PC3 cells were treated with DMSO control or 10 μM mahanine for 1 and 3 days, following which *RASSF1A* expression was assessed by RT-PCR. *GAPDH* was used as an internal control. **C**. PC3 cells were transfected with shRNA for DNMT1, DNMT3A, DNMT3B or scrambled shRNA. Forty-eight hours after transfection, cells were harvested for RT-PCR analyses to assess *RASSF1A* expression. *GAPDH* was used as an internal control. For DNMT3A, two shRNAs were used to confirm the result. **D**. BPH1 cells were transfected with expression vectors of DNMT1, DNMT3A, DNMT3B or empty vector control. Forty-eight hours after transfection cells were collected for RT-PCR analyses to determine *RASSF1A*, *DNMT1, DNMT3A* and *DNMT3B* expression levels. *GAPDH* was used as an internal control.

Our prior work demonstrated the ability of mahanine to restore *RASSF1A* expression in various cancer cell lines, including prostate cancer [25]. To further explore this finding, we analyzed the methylation pattern of the *RASSF1A* promoter using methylation-specific PCR in PC3 and LNCaP cells treated with mahanine for a period of 24 and 72 hours. Expectedly, we observed high amounts of methylated *RASSF1A* promoter in untreated PC3 and LNCaP cells, confirming that *RASSF1A* expression is silenced in prostate cancer cells. However, after 24 hours of mahanine treatment, there was a noticeable increase in the levels of unmethylated *RASSF1A* promoter compared to control; and after 72 hours, this effect was even more dramatic, with higher amounts of unmethylated *RASSF1A* promoter than methylated in the mahanine-treated cells (Figure 1A and Additional file 1: Figure S1A). In order to confirm that the observed decrease in methylation of the *RASSF1A* promoter upon mahanine treatment results in the restoration of its expression in cells, we treated PC3 cells with mahanine for 72 hours and checked the message levels of *RASSF1A* by RT-PCR. The detectable expression of *RASSF1A* after mahanine treatment for 72 hours, but not 24 hours (Figure 1B), correlates well with our PCR data, and confirms the ability of mahanine to restore *RASSF1A* expression by decreasing the methylation of its promoter in prostate cancer cells.

### All three DNMTs possess the ability to silence *RASSF1A* expression

The over-expression DNMTs and the silencing of *RASSF1A* expression by hypermethylation of its promoter in prostate cancer have been well documented [29]. In order to establish the role of the various DNMTs in mediating the methylation of the *RASSF1A* promoter in our system, we down-regulated the expression of *DNMT1, DNMT3A and DNMT3B* in PC3 cells using specific shRNA constructs. Loss of expression of either member of the DNMT family resulted in restoration of RASSF1A expression; however to a lesser extent with the DNMT3A knock-down (Figure 1C). This suggests that all three members of the DNMT family of proteins are involved in the silencing of *RASSF1A* expression, although the degree to which each member is involved varies. Alternatively, we transfected BPH1 cells, in which the native expression of *DNMTs* is almost undetectable, with expression vectors of all three types of the DNMTs (Additional file 1: Figure S1B). The exogenous expression of either member of the *DNMT* family completely inhibited *RASSF1A* expression (Figure 1D), suggesting that either one of the three DNMTs are capable of silencing *RASSF1A* expression in prostate epithelial cells.

### Mahanine decreases nuclear localization of DNMT1 and DNMT3B

**Figure 2:**
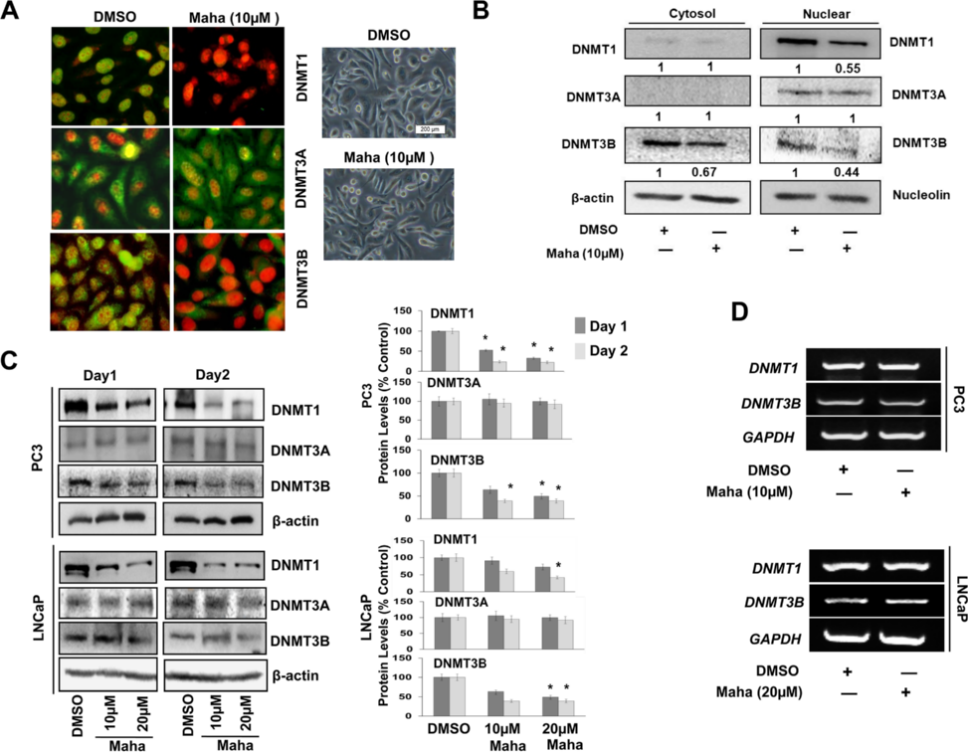
**Mahanine specifically down-regulates DNMT1 and DNMT3B. A**. DNMT1, DNMT3A and DNMT3B cellular localization was visualized by immunofluorescent staining*.* PC3 cells were treated with DMSO (as control) or mahanine (10 μM) for 24 hours, following which they were fixed in methanol, incubated with the indicated antibodies, stained with Alexa Fluor 488-tagged secondary antibodies and counterstained with propidium iodide. Slides were then mounted and examined under a fluorescence microscope. The bright field images of PC3 cells treated with DMSO or mahanine (10 μM) for 24 hours are shown (right panel). **B**. Cytoplasmic and nuclear fractions were separated from PC3 cells treated with DMSO or 10 μM mahanine for 24 hours. The isolated fractions were subjected to Western blot analysis to assess DNMT expression. The fold change in the expression of the respective DNMTs as compared to the control is indicated at the bottom of each immunoblot. Nucleolin and β-actin were used as loading controls for the nuclear and cytoplasmic fractions, respectively. **C**. PC3 and LNCaP cells were treated as indicated with DMSO or mahanine following which cells were lysed and the extracts were subjected to Western blot analysis to detect DNMT1, DNMT3B and DNMT3A protein levels (left). Quantitative estimations of the relative levels of DNMT1, DNMT3A and DNMT3B proteins were determined by densitometric measurements of immunoblots from three independent experiments after normalization with β-actin (right). Columns, mean; bars, SEM. *p < 0.05, significantly different from control. **D**. PC3 and LNCaP cells were treated with DMSO or 10 and 20 μM mahanine, respectively for 24 hours. Subsequently, cells were harvested for RT-PCR analysis to measure *DNMT1* and *DNMT3B* expression*. GAPDH* was used as an internal control.

Our prior work suggests that the inactivation of DNMTs is involved in the mechanism by which mahanine induces the restoration of *RASSF1A* expression in prostate cancer cells [25]. To understand how mahanine mediates the inactivation of DNMTs, we evaluated the effect of mahanine treatment on the cellular localization of the three DNMTs by immunocytochemical staining, since DNMTs must localize to the nucleus to carry out their effects. Our data revealed that DNMT1 is localized exclusively in the nucleus in DMSO-treated, control PC3 (Figure 2A and Additional file 2: Figure S2A) and LNCaP (data not shown) cells. After treatment with mahanine (10μM) for 24 hours, the nuclear levels of DNMT1 were reduced to an undetectable level (Figure 2A and Additional file 2: Figure S2A). DNMT3B appeared to be distributed between the nucleus and cytoplasm in control cells; however, upon treatment with mahanine, its nuclear levels were undetectable, with a noticeable decline in its cytoplasmic staining as well. In comparison with DNMT1 and DNMT3B, the cellular distribution of DNMT3A remained mostly unchanged by mahanine treatment.

To further confirm our immunocytochemical data, PC3 cells were treated in a similar manner with mahanine for 24 hours and nuclear and cytoplasmic fractions were separated from the treated and untreated cells. Our data confirmed that while mahanine decreased DNMT1 levels from the nucleus (~2-fold), and DNMT3B levels in the cytoplasm (~1.5-fold) and in the nucleus (~2.2-fold), it left DNMT3A levels unchanged, suggesting that mahanine modulates the cellular localization and protein levels of DNMT1 and DNMT3B, without affecting DNMT3A (Figure 2B).

### Mahanine down-regulates DNMT1 and DNMT3B, but not DNMT3A protein levels in prostate cancer cells

To determine if mahanine affected the total cellular levels of the DNMTs, we treated androgen receptor-negative PC3 cells and androgen-responsive LNCaP cells with 10μM and 20μM of mahanine for a period of 24 to 48 hours. We observed that while mahanine causes a dose- and time-dependent down-regulation in the protein levels of DNMT1 and DNMT3B in both cell lines, it has no effect on DNMT3A protein levels, which is in agreement with our findings from the immunocytochemical and nuclear-cytoplasmic fractionation experiments (Figure 2C and Additional file 2: Figure S2B).

Next, we sought to determine whether the observed decline in DNMT1 and DNMT3B levels was due to suppression of their gene expression upon mahanine treatment. We measured the message levels of these *DNMT*s as an indicator of the level of their gene expression. We did not detect a noticeable change in the message levels of *DNMT1* and *DNMT3B* in LNCaP or PC3 cells with 20μM and 10μM mahanine treatment, respectively for a period of 24 hours (Figure 2D), suggesting that the decline in DNMT1 and DNMT3B protein levels occurs post-translationally.

### Mahanine-induced depletion of DNMTs is not mediated by caspases

**Figure 3:**
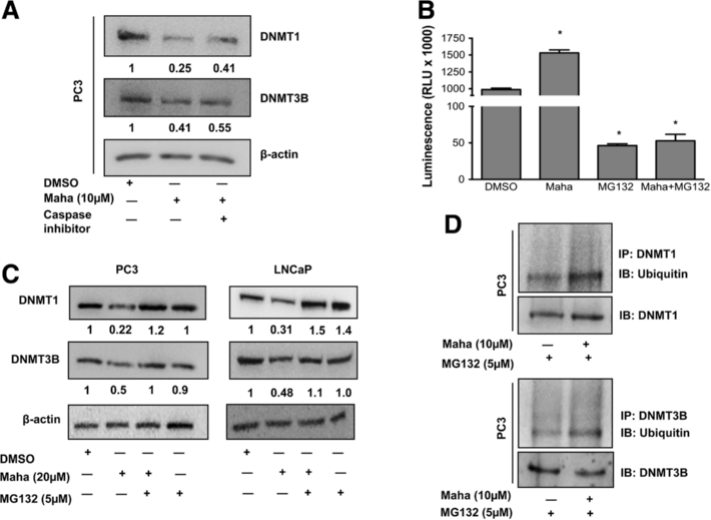
**Mahanine degrades DNMTs via the ubiquitin-proteasomal pathway. A**. PC3 cells were treated with 10 μM mahanine and 20 μM Z-VAD-FMK for 24 hours after which cellular protein lysates were subjected to Western blot analysis to detect DNMT1 and DNMT3B protein levels, β-actin was used as a loading control. **B**. Chymotrypsin-like proteasomal activity was measured in PC3 cells treated as indicated with mahanine and MG132 for 24 hours. Columns, mean; bars, SEM. *p < 0.05, significantly different from DMSO control. **C**. LNCaP and PC3 cells were treated with the indicated doses of mahanine for 24 hours with or without MG132 (5 μM). Cell lysates were analyzed for DNMT1 and DNMT3B expression by Western blot. β-actin was used as a loading control. **D**. PC3 cells were treated with MG132 (5 μM) in the absence and presence of mahanine (10 μM) for 24 hours. Cell lysates were subjected to immunoprecipitation (IP) of DNMT1 or DNMT3B and immunoblotted (IB) for poly-ubiquitin, DNMT1 and DNMT3B.

In a previous publication we demonstrated that high micromolar concentration of mahanine treatment induces apoptosis in prostate cancer cells [30]. Since activation of caspases degrades various proteins within the cell, we wanted to evaluate whether the decline in cellular levels of DNMT1 and DNMT3B upon treatment with mahanine is mediated by a caspase-dependent pathway. To this end, we performed live/dead cell assay and measured the caspase activity in LNCaP and PC3 cells with various doses of mahanine for a period of 24 hours. We did not observe an induction of cell death (Additional file 3: Figure S3A) or caspase activity (Additional file 4: Figure S4A), suggesting that apoptotic pathways had not been induced in the cells within this time frame of mahanine treatment (although growth was significantly arrested as noted in Additional file 5: Figure S5). In addition, we treated PC3 cells with mahanine (10μM) in the absence and presence of a pan-caspase inhibitor (Z-VAD-FMK), and checked the cellular levels of DNMT1 and DNMT3B. The inhibition of caspases did not hinder the ability of mahanine to cause a decline in the levels of these DNMTs, suggesting that mahanine alters their levels by an alternative mechanism, without the involvement of caspases (Figure 3A).

### Mahanine degrades DNMTs via the ubiquitin-proteasomal pathway

Our data so far eliminates the possibility that transcriptional down-regulation and caspase mediated degradation might be the mechanism by which mahanine decreases the levels of DNMT1 and DNMT3B. Next, we sought to determine whether mahanine mediates DNMT depletion through the ubiquitin-proteasomal pathway. Proteasomal degradation generally occurs via three types of enzymatic activities; specifically, chymotrypsin-like, trypsin-like, and caspase-like. Among these, chymotrypsin-like activity is the rate limiting step in the proteasomal degradation process. To this end, we measured proteasomal activation in PC3 cells treated with mahanine in the absence and presence of a proteasome inhibitor, MG132. We found that while mahanine induced chymotrypsin-like proteasomal activity, it caused a decline in the trypsin-like and caspase-like activities of the proteasome (Figure 3B and Additional file 4: Figure S4B).

To determine whether the induction of chymotrypsin-like proteasome activity by mahanine could account for the decline in DNMT levels, PC3 and LNCaP cells were treated with 10μM and 20μM mahanine, respectively for 24 hours in the presence and absence of MG132 (5 μM). While mahanine treatment resulted in a decline in the levels of DNMT1 and DNMT3B in both cell lines, this effect was rescued upon inhibition of the proteasome, suggesting that mahanine causes DNMT degradation via a chymotrypsin-like proteasomal mechanism (Figure 3C). Since proteasomal degradation is preceded by ubiquitination, we immuno-precipitated DNMT1 and DNMT3B from PC3 cells that had been treated with mahanine and MG132, and found an increase in the levels of ubiquitinated DNMTs in the presence of mahanine (Figure 3D). Similarly, in mahanine-treated PC3 cells we observed an increase in total ubiquitination (Additional file 4: Figure S4C). Taken together, our data suggest that mahanine decreases the cellular levels of DNMT1 and DNMT3B by a ubiquitin-proteasome mediated pathway.

### Mahanine down-regulates pAkt levels in PC3 and LNCaP cells

**Figure 4:**
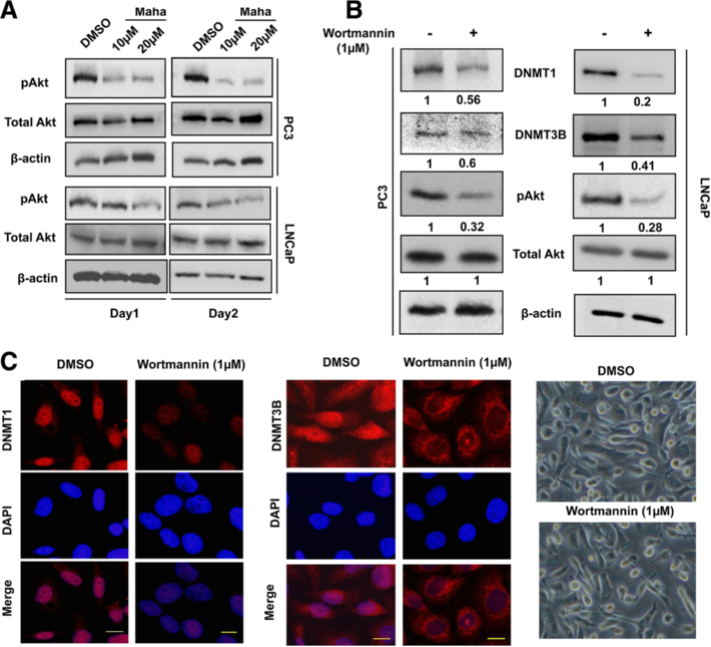
**Mahanine down-regulates pAkt levels and PI3K/Akt inhibitor wortmannin reduces DNMT1 and DNMT3B protein levels. A**. PC3 and LNCaP cells were treated with mahanine as indicated. The cell lysates were subjected to Western blot analysis for phospho Akt (pAkt), total Akt. Β-actin was used as a loading control. **B**. PC3 and LNCaP cells were treated with wortmannin (1 μM) for 24 hours. Cell lysates were subjected to Western blot analysis to measure DNMT1, DNMT3B, pAkt and total Akt levels. β-actin was used as a loading control. The fold change in expression of the respective proteins compared to control is indicated below. **C**. PC3 cells were treated with DMSO or wortmannin (1 μM) for 24 hours. DNMT1 and DNMT3B cellular localization was visualized by immunofluorescent staining*.* After 24h of treatment, cells were fixed in methanol, incubated with the indicated antibodies, stained with Alexa Fluor 594-tagged secondary antibodies and counterstained with DAPI. Slides were then mounted and examined under a fluorescence microscope. The bright field images of PC3 cells treated with DMSO or wortmannin (1 μM) for 24 hours are shown (right panel).

The high activity of the survival kinase Akt in prostate cancer cells is well documented [31,32]. Our previous work has established the ability of mahanine to inactivate Akt signalling in prostate cancer cells, thereby inhibiting its down-stream effects like cellular proliferation and survival [30]. Akt phosphorylation has been found to be one of the major factors responsible for DNMT1 stabilization [33]. To evaluate whether Akt inhibition was involved in DNMT inactivation and degradation brought about by mahanine, we first measured the levels of activated Akt in whole cell lysates of PC3 and LNCaP cells treated with or without mahanine. Expectedly, mahanine treatment decreased phospho-Akt levels in both, PC3 and LNCaP cells, without affecting total Akt expression (Figure 4A), thereby confirming our previous findings.

### PI3K inhibitor, wortmannin degrades DNMT1 and DNMT3B proteins in prostate cancer cells

Phosphoinositide 3-kinase or PI3K is an enzyme which acts directly upstream of Akt and is essential for its activation. To understand the relationship between Akt activation and the stability of DNMTs in prostate cancer cells, PC3 and LNCaP cells were treated with a PI3K inhibitor, wortmannin for 24 hours. As expected, inhibition of PI3K by wortmannin reduced phospho-Akt levels significantly after 24 hours of treatment. Furthermore, the inactivation of Akt was accompanied by a significant down-regulation in DNMT1 protein levels both in PC3 (~ 2-fold) and LNCaP (~ 5-fold) cells (Figure 4B and Additional file 6: Figure S6). A similar trend was observed in the case of DNMT3B, with an approximate 40% and 60% reduction in its levels in PC3 and LNCaP cells, respectively. The decrease in the levels of DNMT1 and DNMT3B after 24 hours of wortmannin treatment was also observed upon immunocytochemical analyses in PC3 cells (Figure 4C). These data confirm that DNMT stability is mediated by Akt activity and a disruption of Akt activation decreases DNMT levels in prostate cancer cells.

### Mahanine treatment disrupts the interaction of pAkt with DNMT1 and DNMT3B in prostate cancer cells

**Figure 5:**
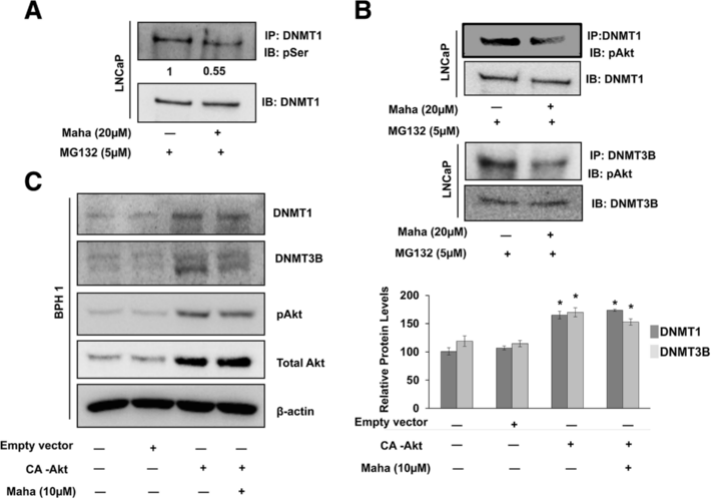
**Mahanine disrupts the interaction of pAkt with DNMT1 and DNMT3B and constitutively active Akt (CA-Akt) stabilizes their cellular levels in the presence of mahanine. A**. and **B**. LNCaP cells were treated with MG132 with or without 20 μM mahanine for 24 hours and the cell homogenates were subjected to co-immunoprecipitation (IP) for DNMT1 or DNMT3B and immunoblotted (IB) for phosphor-serine (pSer), pAkt, total Akt, DNMT1 and DNMT3B. **C**. BPH1 cells were transfected with an empty vector or a constitutively active Akt (CA-Akt) expression vector for 24 hours then were treated with or without mahanine (10 μM) for another 24 hours. Levels of total Akt, pAkt, DNMT1 and DNMT3B proteins were measured through Western blots. β-actin was used as a loading control. Quantitative estimations of relative levels of DNMT1 and DNMT3B proteins were determined by densitometric measurements of immunoblots from three independent experiments after normalization with β-actin. Columns, mean; bars, SEM. *p < 0.05, significantly different from DMSO treated control.

Others have demonstrated that Akt stabilizes DNMT1 by phosphorylating its Ser 143 residue, and the dephosphorylation of this site makes DNMT1 vulnerable to ubiquitin-proteasomal degradation [28]. Our finding that the ubiquitination and proteasomal degradation of DNMT1 and DNMT3B increases in the presence of mahanine prompted us to check whether mahanine interfered with Akt mediated stabilization of these DNMTs. Upon immunoprecipitation of DNMT1 from LNCaP cells treated with mahanine and MG132 to prevent degradation, we observed a two-fold decline in serine phosphorylation of DNMT1 in the presence of mahanine, suggesting that mahanine is involved in modulating the phosphorylation of DNMT1 (Figure 5A). Furthermore, we observed a decline in the interaction of phospho-Akt with DNMT1 and DNMT3B in the presence of mahanine, indicating that mahanine treatment disrupts the interaction of activated Akt with these DNMTs (Figure 5B). These data indicate that mahanine modulates stabilizing post-translational modifications, such as serine phosphorylation, of DNMT1. In addition, the interaction of a stabilizing kinase Akt with DNMT1 and DNMT3B is inhibited in the presence of mahanine, suggesting that the mechanism of degradation of DNMTs by mahanine could involve Akt inhibition.

### Constitutively active Akt (CA-Akt) rescues mahanine mediated DNMT1 and DNMT3B degradation in BPH1 cells

To determine whether mahanine induced DNMT1 and DNMT3B degradation occurs via the inhibition of Akt activation, BPH1 cells were transfected with a constitutively active Akt expression construct (CA-Akt) or empty vector following which the cells were treated with or without mahanine (10μM) for 24 hours. BPH1 cells are known to natively express low levels of DNMT1, DNMT3B and Akt. The presence of CA-Akt expectedly induced DNMT1 and DNMT3B levels compared to the control and empty vector transfected. Surprisingly, this induction was maintained in the presence by mahanine, indicating that when Akt is constitutively active, mahanine cannot carry out proteasomal degradation of DNMTs (Figure 5C). This clearly demonstrates that the degradation of DNMTs by mahanine stems from its ability to inactivate Akt.

## Discussion

The silencing of the *RASSF1A* gene has been associated with advanced stages of prostate cancer [22]. Our prior work demonstrated that mahanine possesses the ability to restore *RASSF1A* expression in several different cancer cell lines, including androgen-responsive LNCaP and androgen-receptor negative PC3 prostate cancer cells, which have been derived from metastases to the lymph node and bone, respectively [25]. However, the scope of that report did not include defining a molecular mechanism to explain the re-expression of *RASSF1A* upon mahanine treatment, although it did show a decline in total DNMT activity in the presence of mahanine [25]. Our current work stems from these findings, and in this report we establish a molecular mechanism for the inhibition of DNMT signalling by mahanine; furthermore, we delineate the correlation between DNMT and *RASSF1A* expression in prostate cancer cells.

Our data demonstrate that the re-expression of *RASSF1A* upon mahanine treatment is a time sensitive event; while the demethylation of the *RASSF1A* promoter is apparent after 24 hours of mahanine treatment, *RASSF1A* expression can only be detected after 72 hours of mahanine treatment. This shows that the partial de-methylation of the *RASSF1A* promoter which occurs after 24 hours of mahanine treatment is not enough to restore its expression; the promoter region must be demethylated to a greater extent, which is only achieved approximately 72 hours following mahanine treatment. This suggests that mahanine could modulate the activities of certain factors involved in the maintaining the methylation pattern of the *RASSF1A* promoter region, which accounts for the time lag between mahanine treatment and *RASSF1A* re-expression. Since DNMT1, DNMT3A and DNMT3B are known to be involved in de novo methylation and the maintenance of methylation patterns of genes, we investigated the levels of expression of all three members of the DNMT family. Our data clearly indicates that mahanine treatment causes a decline in the levels of DNMT1 and DNMT3B, without affecting the levels of DNMT3A, in both LNCaP and PC3 prostate cancer cells. Interestingly, the time frame within which mahanine down-regulates DNMT1 and DNMT3B in LNCaP and PC3 cells correlates well with the re-expression of *RASSF1A* in these cell types, suggesting that mahanine could mediate *RASSF1A* re-expression via DNMT inhibition. Furthermore, these results indicate that mahanine selectively modulates the cellular levels of certain DNMTs, without ubiquitously down-regulating the levels of all members of the DNMT family. This data is further supported by our findings that the knock-down or over-expression of either member of the *DNMT* family is sufficient to restore or inhibit the expression of *RASSF1A* in PC3 or BPH1 cells, respectively; however to a lesser extent with DNMT3A. Therefore, mahanine selectively degrades the two DNMTs which appear to most strongly inhibit *RASSF1A* expression in PC3 prostate cancer cells. The ability of mahanine to selectively target DNMT1 and DNMT3B clearly differentiates it from other known DNMT inhibitors like 5-aza cytidine and 5-aza-2′-deoxycitidine, which ubiquitously bind to and irreversibly inhibit all members of the DNMT family. Therefore, anti-cancer agents like mahanine which selectively targets DNMT1 and DNMT3B could be beneficial in prostate cancer therapy.

While mahanine causes degradation of DNMT1 and DNMT3B by inducing the chymotrypsin-like activity of the proteasome, it is interesting to note that it does not potentiate the trypsin- and caspase-like activities of the proteasome. The decline in these particular enzymatic activities of the proteasome upon mahanine treatment could be to compensate for the significant induction of the chymotrypsin-like activity upon mahanine treatment and thereby ensure that the overall proteasomal activity is balanced and cellular homeostasis is undisturbed.

The activity of survival kinases such as Akt is known to be highly up-regulated in prostate cancer, which correlates with the high abundance of DNMTs in prostate cancer cells, as Akt is involved in the stabilization of DNMT1, and possibly other DNMTs, via site-specific phosphorylation on Ser/Thr residues. Interestingly, the degradation of DNMTs by mahanine is dependent on its ability to inhibit Akt activity; when Akt is constitutively active mahanine treatment does not result in proteasomal degradation of DNMT1 and DNMT3B. A recent report demonstrated that Akt phosphorylates the Ser143 residue of DNMT1 and thereby increases its stability [28]. However, no such stabilizing phosphorylation events have been described to date for DNMT3B. Our data clearly indicates that Akt is involved in stabilizing not only DNMT1, but also DNMT3B, since constitutively active Akt renders both DNMTs resistant to proteasomal degradation induced by mahanine. The mechanism by which Akt imparts increased stability to DNMT3B remains to be explored. In addition, our immunoprecipitation data shows that mahanine causes a striking decrease in the overall serine phosphorylation of DNMT1, suggesting that mahanine might modulate the phosphorylation status of DNMT1 on more than one serine residue, via the inactivation of other Ser/Thr kinases, in addition to Akt. It will be interesting to further explore specific phospho-sites involved in imparting stability to DNMT1 and DNMT3B and the kinases responsible for this phosphorylation.

## Conclusions

**Figure 6:**
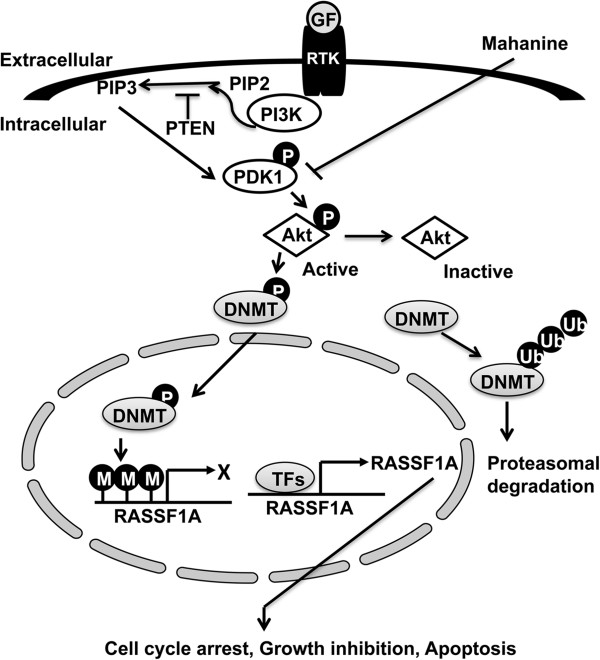
**Mahanine restores *RASSF1A *expression by degrading DNMTs via Akt.** Prostate cancer cells express high levels of activated Akt, which phosphorylates and stabilizes DNMT1 and DNMT3B against proteasomal degradation. DNMTs enter the nucleus and methylate the promoter of *RASSF1A* gene to silence the expression of *RASSF1A*. Treatment of mahanine inhibits PDK1 and thereby prevents activation of Akt, which in turn compromises the stability of DNMTs, increases their ubiquitination and induces proteasomal degradation. In the absence of DNMT1 and DNMT3B, the *RASSF1A* promoter is demethylated and its expression is restored in prostate cancer cells. GF: Growth factor; RTK: Receptor tyrosine kinase; TFs: Transcription factors; P: Phosphorylated; M: Methylated; Ub: Ubiquitinated.

In summary, the data presented in this report indicates that mahanine selectively degrades DNMT1 and DNMT3B via the ubiquitin-proteasomal pathway in a manner dependent on the inactivation of Akt signaling. The degradation of DNMT1 and DNMT3B prompts the demethylation of the promoter of the silenced tumour suppressor gene *RASSF1A*, leading to the restoration of its expression in prostate cancer cells (Figure 6). Therefore, mahanine could potentially be used in the therapy of advanced prostate cancer in men when *RASSF1A* expression is silenced.

## Methods

### Mahanine purification, cell culture, and cell transfection

Mahanine was purified from the leaves of *Murraya koenigii* as described in our previous published report [30]. Human prostate cancer cell lines, PC3 and LNCaP were obtained from American Type Culture Collection (Manassas, VA). Cells were grown in IMEM (Gibco Life Technologies, Grand Island, NY) containing 10% fetal bovine serum (FBS), 2 mM glutamine, 100 u/ml penicillin G sodium, and 100 mg/ml streptomycin sulfate at 37°C and 5% CO_2_[30]. BPH1 cells were obtained from Dr. Simon W. Hayward of Vanderbilt University and cultured in the same medium as mentioned above.

PC3 cells were transfected with DNMT shRNAs (OriGene Technologies Inc., Rockville, MD) and RASSF1A expression vector [34]. BPH1 cells were transfected with 1μg/ml of DNMT expression vectors (OriGene) or constitutively active Akt (CA-Akt) #17-254 (Upstate Biotechnology, Lake Placid, NY) plasmids using Genjet vII reagent (Signagen, Gaithersburg, MD). Forty-eight hours after transfection, cells were harvested for RT-PCR analyses.

Live and dead cells were determined according to the manufacturers’ instructions (Cell Viability/Cytotoxicity Assay kit, Biotium Inc. Hayward, CA)

### Western blot analysis

Western blot analysis was performed according to our earlier published report [35]. In brief, After 24 hours of mahanine, wortmannin, and MG132 treatment, cellular protein extracts were prepared from PC3 and LNCaP cells after various treatments of mahanine, wortmannin, and MG132. Fifty micrograms of proteins were resolved on 8% SDS–PAGE and transferred onto nitrocellulose membranes (Amersham Hybond-ECL), and immunoblotted with primary antibodies (DNMT1 #2788-1, DNMT3A #3116-1, DNMT3B #2601-1 and poly-Ubiquitin #6686-1 antibodies were purchased from Epitomics, Burlingame, CA; phosphorylated-Akt S473 #4060 and total Akt #4685 were purchased from Cell Signaling Technologies, Beverly, MA; β-actin #Sc47778 antibody was purchased from Santa Cruz Biotechnology, Inc., Santa Cruz, CA; and phospho-serine #612546 antibody was obtained from BD Biosciences, San Jose, CA), overnight at 4°C. Immunoreactive protein bands were detected with horseradish peroxidise-conjugated secondary antibodies (Santa Cruz) and with enhanced chemiluminescence system (Santa Cruz) according to the manufacturer's instructions. Band intensities (pixel densities) were quantified by ImageJ software (National Institutes of Health, Bethesda, MD). The preparation of nuclear extracts and immunoprecipitations were performed according to our previously published methods [36].

### Immunofluorescence staining

Immunofluorescence staining was performed according to our previously published report [37]. PC3 cells were plated onto ECL-coated chamber slides and were treated with mahanine, wortmannin, or DMSO as controls. The cells were fixed in −20°C methanol, air-dried, and re-hydrated in PBS. After blocking (0.2% BSA) cells were incubated with the primary antibodies (DNMT1, DNMT3A or DNMT3B #Sc376043 from Santa Cruz Biotechnology) overnight at 4°C, followed by incubation with the Alexa Fluor 488 or 594 conjugated secondary antibodies (Molecular probes/Invitrogen, Carlsbad, CA) for 1 hour. Slides were washed, counterstained with DAPI or with propidium iodide, mounted and viewed under a fluorescent Olympus BX microscope (Olympus Corp, Tokyo, Japan). Images were captured at the same magnification (20X) and then imported into Adobe Photoshop.

### *RASSF1A* promoter methylation assay

The methylation status of *RASSF1A* promoter (Amplicon location: 17881–18115) was determined by Methylation specific primers according to the previously published protocol [38,39] with some modifications. In brief, PC3 cells were treated with or without mahanine, and the bisulfite modified DNA was isolated using EZ DNA Methylation™ Kit procured from Zymo research (Irvine, CA). The PCR reaction was conducted with 4μl of bisulfite-modified DNA with methylated-specific primers for *RASSF1A* (forward primer 5’- GGG TTT TGC GAG AGC GCG -3’, reverse primer 5’- GCT AAC AAA CGC GAA CCG -3’) at the annealing temperature of 54°C. For unmethylated *RASSF1A* (forward primer 5’- GGT TTT GTG AGA GTG TGT TTA G -3’, reverse primer 5’- CAC TAA CAA ACA CAA ACC AAA C -3’) annealing temperature was 47.5°C. After amplification, PCR products were separated on agarose gel and visualized by ethidium bromide fluorescence using the Fuji LAS-1000 Imager.

### Reverse transcriptase-polymerase chain reaction (RT-PCR)

RNA was extracted from PC3 and LNCaP cells with TRIzol solution as suggested by the manufacturer (Invitrogen, Carlsbad, CA) and genes of interest were amplified using 0.5-1 μg of total RNA using Verso 1-Step RT-PCR kit purchased from Thermo Scientific (Waltham, MA). Human-specific primers were designed using the Primer Quest program and purchased from Integrated DNA Technologies, Inc (Coralville, IA). The primer sequences are: DNMT1 forward primer 5- GTGAGGACATGCA GCTTTCA −3, reverse primer 5- TGCTGCCTTTGATGTAGTCG-3, DNMT3A forward primer 5’-TGAGAGTG ACACTGCCAAGC-3’, reverse primer 5’- CAGCAGATGGTG CAGTAGGA-3’; DNMT3B forward primer 5’-TTTGGCCACCTTCAATAAGC-3’, reverse primer 5’- GGCAACATC TGAAGCCATTT-3’; GAPDH forward primer 5-CCACCCATGGCAAATT CCATGGCA-3, reverse primer 5-TCTAGACGGCAGGTCAGGTCCACC-3’. Glyceraldehyde-3-phosphate dehydrogenase (GAPDH) and RASSF1A primer sequences have been previously published [25]. PCRs were initiated at 47°C for 30 min then 95°C for 1min followed by 30 cycles of 95°C for 1 min, 1 min annealing temperature, 72°C for 1 min, and final extension at 72°C for 3 min. After amplification, PCR products were separated on 1.5% agarose gels and visualized by ethidium bromide fluorescence using the Fuji LAS-1000 Imager.

### Statistical analyses

All data were derived from at least three independent experiments and statistical analyses were conducted by using one-way analysis of variance (ANOVA) followed by the Dunnett’s post-test with an assigned confidence interval of 95%. Values were presented as means ± SEM. *p*-value < 0.05 was considered significant.

## Competing interests

The authors declare that they have no competing interests.

## Authors’ contributions

SA is responsible for the execution, data interpretation, data analyses and drafting of the manuscript. KA contributed to experimental design, conducting various experiments for the revision and the editing of the manuscript. SJ performed several RT-PCR experiments. GB, PR, NB and SB were responsible for the purification of mahanine. PB directed the experimental design and provided insight for experimental execution, and editing the manuscript and figures. All authors have read and approved the final manuscript.

## Supplementary Material

Additional file 1: Figure S1 Mahanine demethylates RASSF1A promoter (A) LNCaP cells were treated with 10 μM mahanine for 3 days. Methylation-specific PCR was performed to detect the methylated (M) and un-methylated (UM) status of *RASSF1A* promoter. (B) BPH1 cells were transfected with DNMT1, DNMT3A and DNMT3B following which the expression levels of the respective DNMTs were assessed by Western blotting. Click here for file

Additional file 2: Figure S2 Mahanine selectively alters DNMT1 and DNMT3B but not DNMT3A. (A) DNMT1, DNMT3A and DNMT3B expression was examined by *immunofluorescent* staining after 24 hours of treatment with DMSO or mahanine (10 μM). (B) BPH1 cells were transfected with DNMT1, DNMT3A and DNMT3B and treated with mahanine (10 μM) for 48 hours following which the expression levels of the respective DNMTs were assessed by Western blotting. Click here for file

Additional file 3: Figure S3 Mahanine does not induce caspase activity. (A) Live and dead staining of LNCaP (upper panel) and PC3 (lower panel) cells cultured for 24 hours with or without mahanine at the indicated doses. The live cells stained with calcein AM appeared green and the dead cells stained with EthD-III appeared red. (B) Bright field images of LNCaP and PC3 cells treated with the indicated dose of mahanine for 24 hours. Click here for file

Additional file 4: Figure S4 Mahanine does not induce trypsin-like or caspase-like proteasomal activity. (A) LNCaP cells were treated with DMSO (as control) or the indicated doses of mahanine for 24 hours and caspase-3/7 activities were measured in cell lysates using fluorescence assay kits. Data are representative of four independent experiments. Columns represents mean, error bars represent SEM. (B) Chymotrypsin-like and caspase-like proteasomal activities were assayed subsequent to incubation with mahanine (10 μM) with or without MG132 (5 μM) for 24 hours. Data are representative of four independent experiments. Columns, mean; bars, SEM. *p < 0.05, significantly different from control.(C) PC3 cells were treated with DMSO or mahanine (10 μM) for 24h and subjected to Western blot analysis for ubiquitination. Click here for file

Additional file 5: Figure S5 Mahanine decreased cell growth. LNCaP and PC3 cells were treated with indicated doses of mahanine for 24-48 hours following which cell viability was assessed by MTT assay. Click here for file

Additional file 6: Figure S6 Akt inhibitor CCT128930 reduces DNMT1 and DNMT3B protein levels. PC3 and LNCaP cells were treated with CCT128930 (10 μM) for 24 hours. Cell lysates were subjected to Western blot analysis to measure DNMT1, DNMT3B and pGSK levels. β-actin was used as a loading control. Click here for file
