# Supplementary figures and images for: Mahanine restores RASSF1A expression by down-regulating DNMT1 and DNMT3B in prostate cancer cells
Source: Mol Cancer. 2013 Aug 30;12:99. doi: 10.1186/1476-4598-12-99 (PMC3851847; doi:10.1186/1476-4598-12-99)

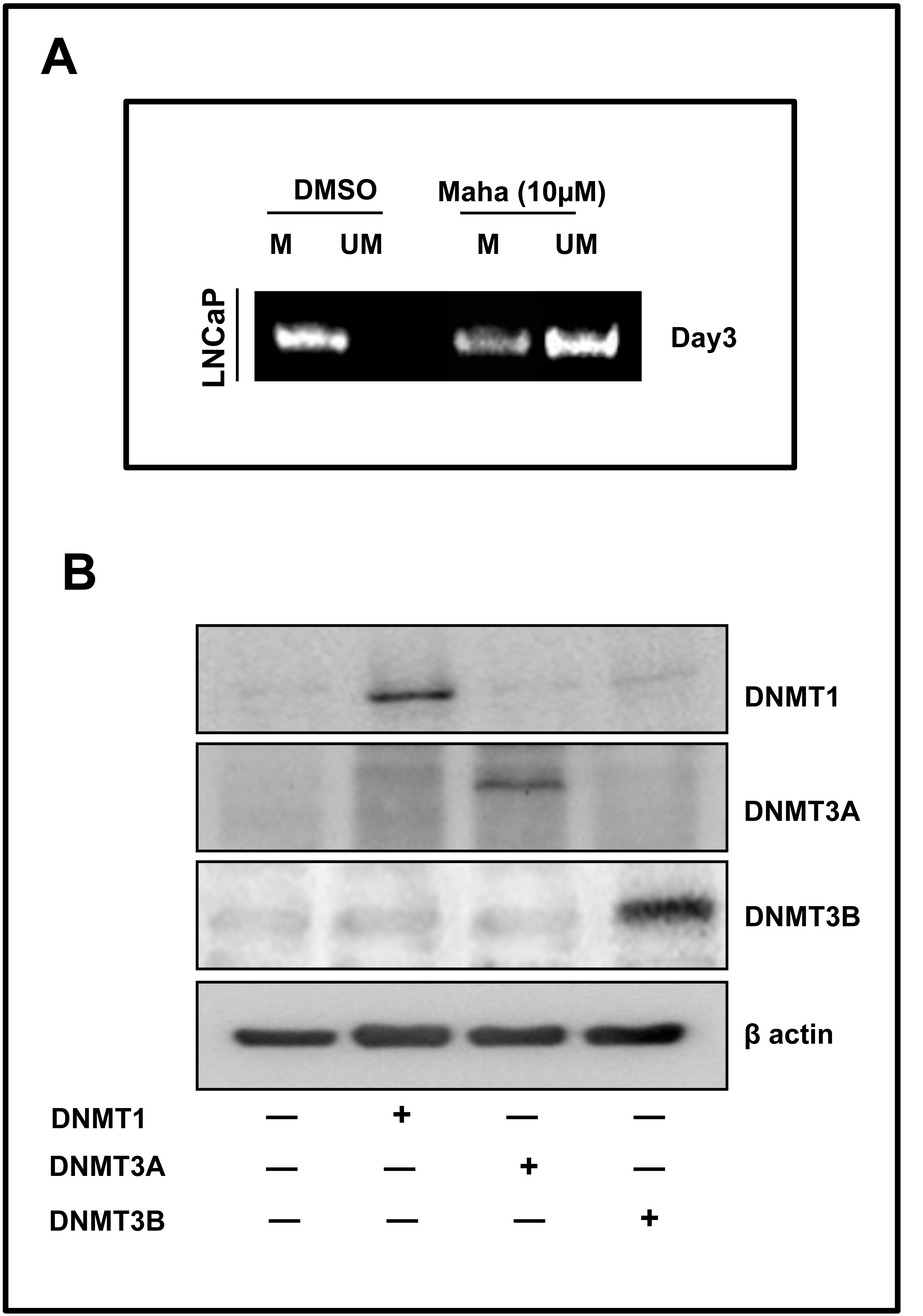

Supplement: Additional file 1: Figure S1 — Mahanine demethylates RASSF1A promoter (A) LNCaP cells were treated with 10 μM mahanine for 3 days. Methylation-specific PCR was performed to detect the methylated (M) and un-methylated (UM) status of RASSF1A promoter. (B) BPH1 cells were transfected with DNMT1, DNMT3A and DNMT3B following which the expression levels of the respective DNMTs were assessed by Western blotting. [file 1476-4598-12-99-S1.png]

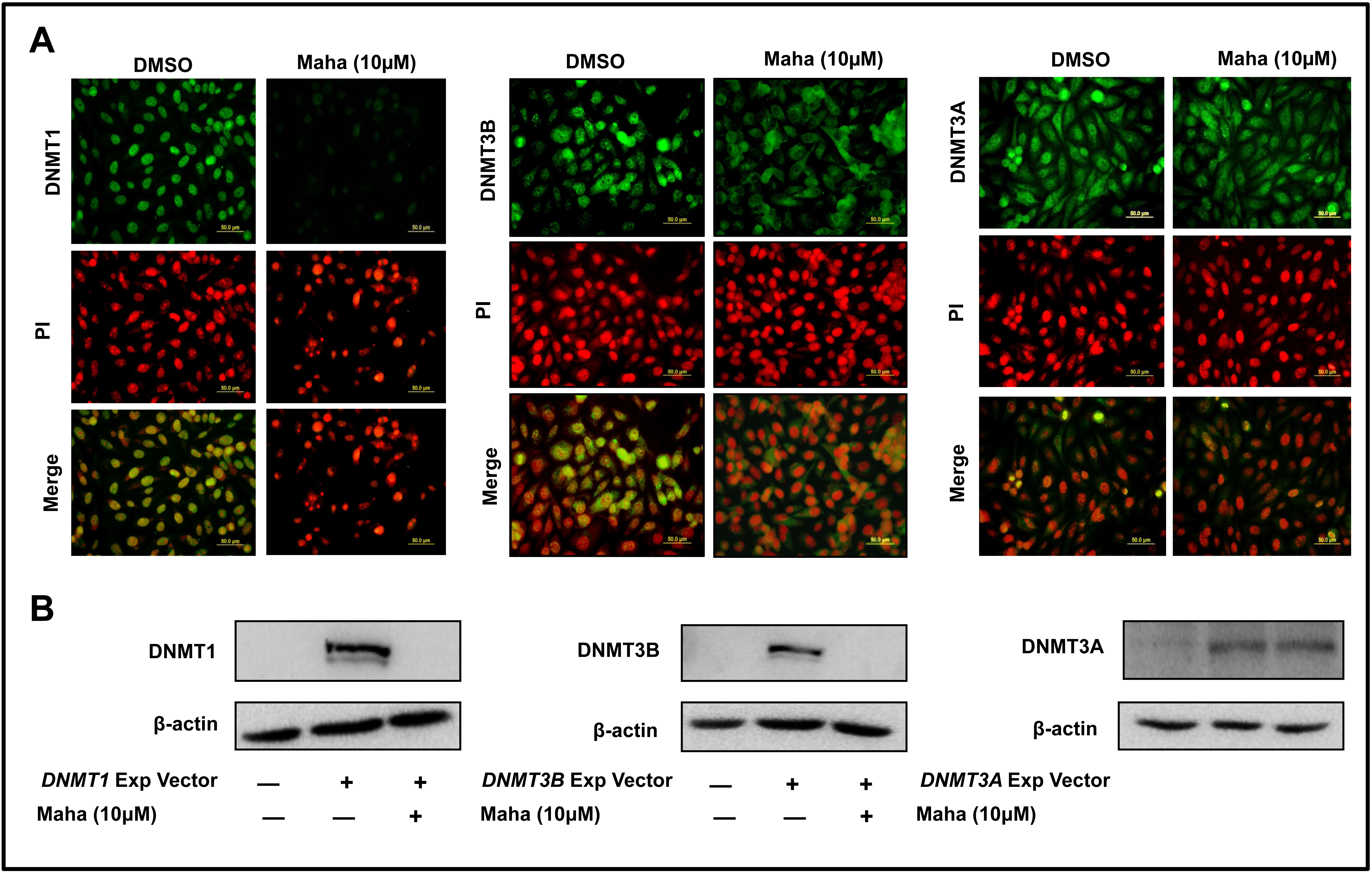

Supplement: Additional file 2: Figure S2 — Mahanine selectively alters DNMT1 and DNMT3B but not DNMT3A. (A) DNMT1, DNMT3A and DNMT3B expression was examined by immunofluorescent staining after 24 hours of treatment with DMSO or mahanine (10 μM). (B) BPH1 cells were transfected with DNMT1, DNMT3A and DNMT3B and treated with mahanine (10 μM) for 48 hours following which the expression levels of the respective DNMTs were assessed by Western blotting. [file 1476-4598-12-99-S2.png]

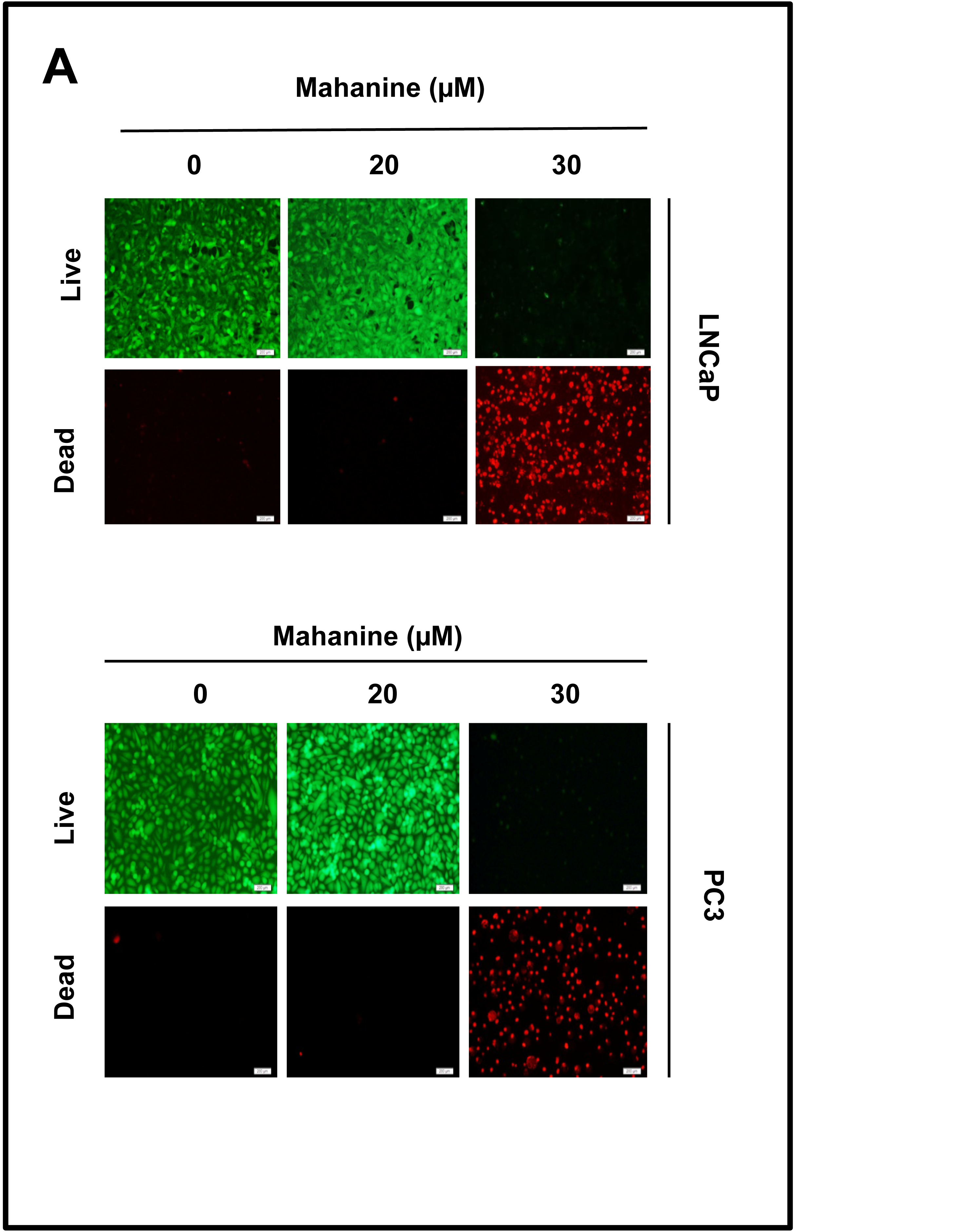

Supplement: Additional file 3: Figure S3 — Mahanine does not induce caspase activity. (A) Live and dead staining of LNCaP (upper panel) and PC3 (lower panel) cells cultured for 24 hours with or without mahanine at the indicated doses. The live cells stained with calcein AM appeared green and the dead cells stained with EthD-III appeared red. (B) Bright field images of LNCaP and PC3 cells treated with the indicated dose of mahanine for 24 hours. [file 1476-4598-12-99-S3.png]

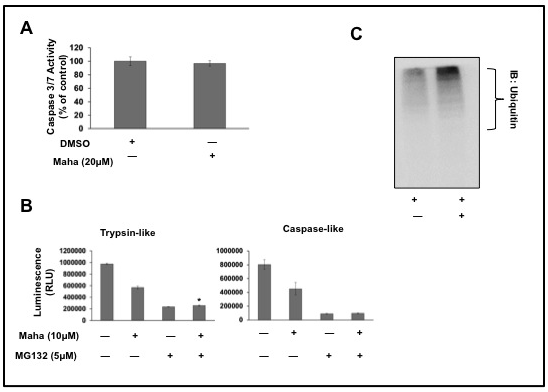

Supplement: Additional file 4: Figure S4 — Mahanine does not induce trypsin-like or caspase-like proteasomal activity. (A) LNCaP cells were treated with DMSO (as control) or the indicated doses of mahanine for 24 hours and caspase-3/7 activities were measured in cell lysates using fluorescence assay kits. Data are representative of four independent experiments. Columns represents mean, error bars represent SEM. (B) Chymotrypsin-like and caspase-like proteasomal activities were assayed subsequent to incubation with mahanine (10 μM) with or without MG132 (5 μM) for 24 hours. Data are representative of four independent experiments. Columns, mean; bars, SEM. *p < 0.05, significantly different from control.(C) PC3 cells were treated with DMSO or mahanine (10 μM) for 24h and subjected to Western blot analysis for ubiquitination. [file 1476-4598-12-99-S4.png]

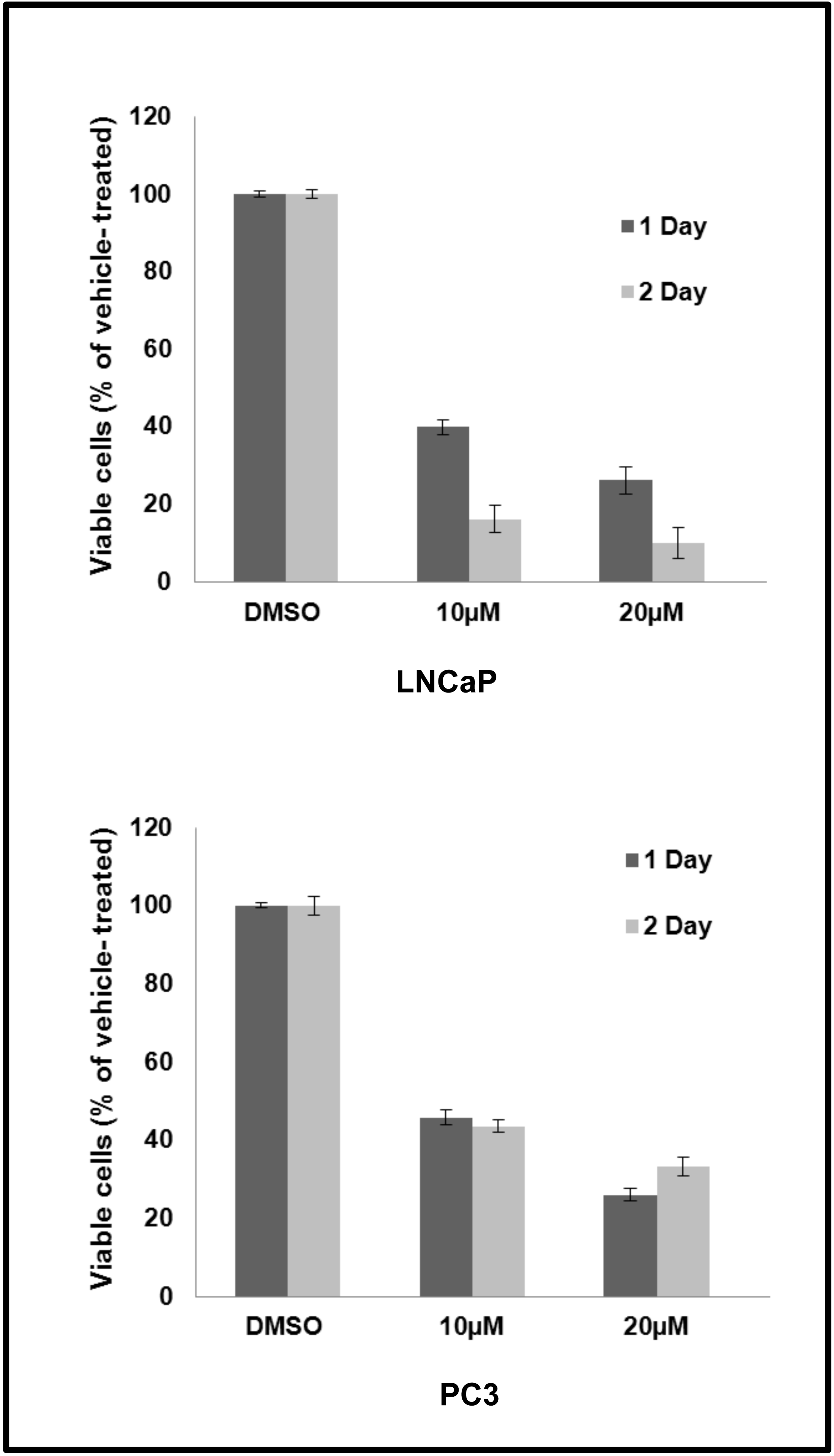

Supplement: Additional file 5: Figure S5 — Mahanine decreased cell growth. LNCaP and PC3 cells were treated with indicated doses of mahanine for 24-48 hours following which cell viability was assessed by MTT assay. [file 1476-4598-12-99-S5.png]

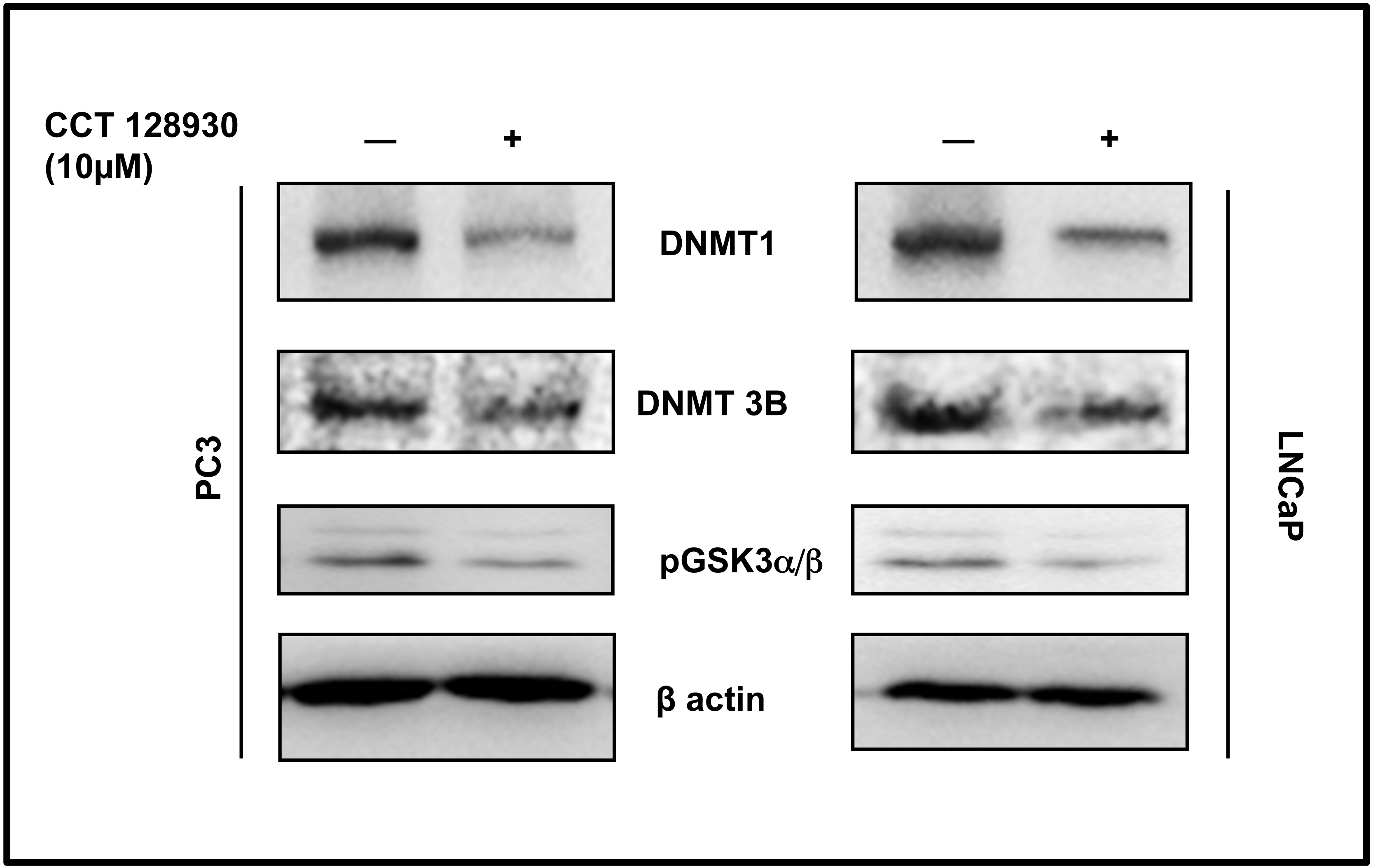

Supplement: Additional file 6: Figure S6 — Akt inhibitor CCT128930 reduces DNMT1 and DNMT3B protein levels. PC3 and LNCaP cells were treated with CCT128930 (10 μM) for 24 hours. Cell lysates were subjected to Western blot analysis to measure DNMT1, DNMT3B and pGSK levels. β-actin was used as a loading control. [file 1476-4598-12-99-S6.png]
